# Impact of bile duct stenting on the management of symptomatic choledocholithiasis: a retrospective multicenter analysis

**DOI:** 10.3389/fsurg.2025.1630416

**Published:** 2025-07-23

**Authors:** Artur Rebelo, Marie L. Tischer, Jonas Rosendahl, Jens Walldorf, Tawfik Mosa, Jörg Kleeff, Johannes Klose

**Affiliations:** ^1^ Department of Visceral, Vascular and Endocrine Surgery, Martin Luther University Halle-Wittenberg, Halle (Saale), Germany; ^2^ Department of Internal Medicine-I, Martin Luther University Halle-Wittenberg, Halle (Saale), Germany; ^3^ Department of Operative Medicine, Carl-von-Basedow-Hospital Saalekreis, Merseburg, Germany

**Keywords:** surgery, ERCP, cholecysititis, cholecystectomy, stent

## Abstract

**Objective:**

Choledocholithiasis (CDL) can lead to various complications and requires treatment approaches for both biliary tract clearing and cholecystectomy. This study aims to characterize CDL patients, evaluate treatment strategies, assess associated complications, and explore economic impacts.

**Methods:**

We conducted a retrospective analysis of 112 patients between 2016 and 2021 at two centers. We performed a descriptive analysis comparing outcomes of patients undergoing ERCP with and without bile duct stenting. Univariate and multivariable analyses were used to identify factors related to complications.

**Results:**

Bile duct stenting was associated with significantly higher complication rates (52.4%) compared to the group without stenting (26.5%) (*p* = 0.006). Factors influencing stent implantation included prior abdominal surgeries (OR = 03.51, *p* = 0.02), cholangitis at admission (OR = 03.02, *p* = 0.032), and bile duct diameter (OR = 01.16, *p* = 0.057). The overall median length of stay was longer for patients with stenting (19 days) compared to those without (11 days) (*p* < 0.001). Finally, reimbursements were higher for patients with stenting. Reimbursement for complicated courses was higher than for those without, independent of initial bile duct stenting (with stent *p* = 0.006, without stent *p* = 0,003).

**Conclusion:**

Bile duct stenting during CDL management is associated with higher complication rates, longer hospital stay, and increased costs. These associations may reflect both clinical severity at baseline and procedural sequencing. A more restrictive placement of biliary stents might be advisable.

## Introduction

In the Western world, approximately 15% of the population is affected by cholelithiasis (CL) (1–5), with higher proportion in elderly (5, 6). Among patients with CL, 5%–15% also develop choledocholithiasis (CDL) (2–5, 7). Conversely, nearly all (95%) patients with CDL also have CL (8, 9). If the demographic changes and the increase in metabolic syndrome continue, the prevalence of CL is expected to rise further (7, 10–12).

Current international guidelines recommend the removal of bile duct stones followed by laparoscopic cholecystectomy (CCE) for the treatment of CDL (13). The procedure for secondary CDL is therefore two-stage and is also known as therapeutic splitting (3, 5, 14). The goal of the first intervention is the removal of gallstones from the bile duct. The preferred method for this is endoscopic retrograde cholangiopancreatography (ERCP) (2, 3, 15, 16). Following stone removal, a plastic stent may be placed in the bile duct. Stenting is a method for draining the bile ducts in patients with incomplete stone removal, cholangitis, high complication risk, or as a bridging option until later treatment (3, 12, 13, 17–20). The stent can facilitate the reduction, fragmentation, and elimination of large bile duct stones. A biofilm can form within the stent, which can lead to stent occlusion and promote infections. For this reason, regular stent changes are recommended every three to six months (13, 18–21).

The optimal timing of CCE after ERCP is the subject of current research. Several guidelines provide recommendations for the management of CDL and the timing of cholecystectomy after ERCP. The S3 guideline from Germany suggests performing CCE within 72 h after a preoperative ERCP in cases of gallstones (3). Similarly, the European Association for the Study of the Liver (EASL) recommends CCE within 72 h following ERCP in its 2016 guideline on gallstones (2). In contrast, the European Society of Gastrointestinal Endoscopy (ESGE) advises CCE within 14 days post-ERCP, as per its 2019 guideline (20). The American Society for Gastrointestinal Endoscopy (ASGE) guideline, however, does not specify a particular interval for CCE following ERCP, leaving the decision to perform ERCP either pre- or postoperatively to the discretion of the clinician (13). Meanwhile, the National Institute for Health and Care Excellence (NICE) guidelines, updated in 2023, do not define a fixed time interval for CCE after ERCP and suggest that randomized controlled trials (RCTs) should be conducted to determine the optimal timing (1). Finally, the Tokyo Guidelines (TG18), while providing recommendations for cholecystitis management, advise performing CCE after 72 h (within one week) for acute cholecystitis but do not provide specific recommendations for CDL management (1, 2, 13, 20, 22).

The aim of this study is to explore differences in the management of CDL. Additionally, the study will examine the outcomes of patients with CDL, with a particular focus on potential complications associated with CDL and economic implications.

## Methods

This study was conducted in accordance with the STROBE guidelines (Strengthening the Reporting of Observational Studies in Epidemiology) to ensure transparent and thorough reporting of the methodology (23).

Ethical approval for the study was obtained from the Ethics Committee of the Medical Faculty of the Martin Luther University Halle-Wittenberg (reference number 2021-201).

We performed a retrospective, anonymized data collection and exploratory analysis of all cases of symptomatic choledocholithiasis (CL) treated between January 1, 2016, and December 31, 2021, at the University Hospital Halle (Saale) (UKH) and the Carl-von-Basedow-Hospital Saalekreis gGmbH (CvBK). The data sources included medical documentation and physician reports, which provided information on patient history, physical examination findings, clinical course including any complications, as well as results from ERCP (endoscopic retrograde cholangiopancreatography) and endosonography, surgical reports, and blood sample laboratory values.

We searched the hospital databases for cases using pre-determined operation and procedure codes (OPS) and ICD-10-GM for the encoding of diagnoses (24). The study population included adult patients with documented choledocholithiasis who underwent the aforementioned procedures. Patients were excluded if the cholecystectomy was performed as part of other abdominal surgeries or if other conditions were responsible for the symptoms and procedures. Additionally, minors, patients undergoing cholecystectomy in the context of malignant diseases, those without CDL diagnosis, or those with insufficient data were excluded. Patients with CDL presenting after a prior cholecystectomy were also excluded to avoid potential confounding factors such as stone migration or secondary stone formation.

All complications that occurred during the course of treatment, understood as deviations from the optimal outcome, were considered. Given the retrospective nature of the study, the complications (such as post-ERCP pancreatitis, cholangitis, and cholangiosepsis) were solely defined based on the documentation provided by the treating physicians. No distinction was made between the possible causes. The timing of the complications was documented as either preoperative or postoperative. In this study, both postoperative complications and post-interventional complications, i.e., those occurring after ERCP (preoperative complications), were recorded.

Complications were categorized as preoperative (occurring after ERCP and before cholecystectomy) or postoperative (occurring after cholecystectomy). The attribution was based on the timing of the documented clinical event in relation to the procedural dates, as recorded in the medical records. In cases where attribution was ambiguous (e.g., when ERCP and cholecystectomy were performed during the same admission), classification was based on the assessment of the treating physician as documented in the discharge summary.

For statistical data analysis, we used the IBM® SPSS Statistics 28. Categorical variables were presented as absolute and relative numbers. Statistical significance was tested using Chi-square tests for expected cell frequencies greater than 5 or Fisher's exact test for expected frequencies below 5. Continuous variables were reported as medians and interquartile ranges (Q1–Q3). Normal distribution of continuous variables was assessed with the Kolmogorov–Smirnov test. Statistical significance was tested using the Student's *t*-test for normally distributed data and the Mann–Whitney *U*-test for non-parametric data. The Kruskal–Wallis test was applied for non-parametric data with more than two groups.

Potential risk factors for the occurrence of complications were identified using multivariable analysis through binary logistic regression. The model structure was based on the approach described by Hosmer and Lemeshow in 2013 (25). Correlations between categorical variables were evaluated using Phi for 2 × 2 tables and Cramer's V for variables with more than two categories (26).

A *p*-value of less than 0.05 was considered statistically significant. Due to the retrospective study design, missing values resulted in the total number for the affected category being adjusted if needed.

To assess potential financial outcomes for the healthcare system, the BWR (relative cost weight) was analyzed. Furthermore, we analyzed hospital revenues in relation to stenting, considering any complications, to explore a potential impact on the payments received.

## Results

**Figure 1:**
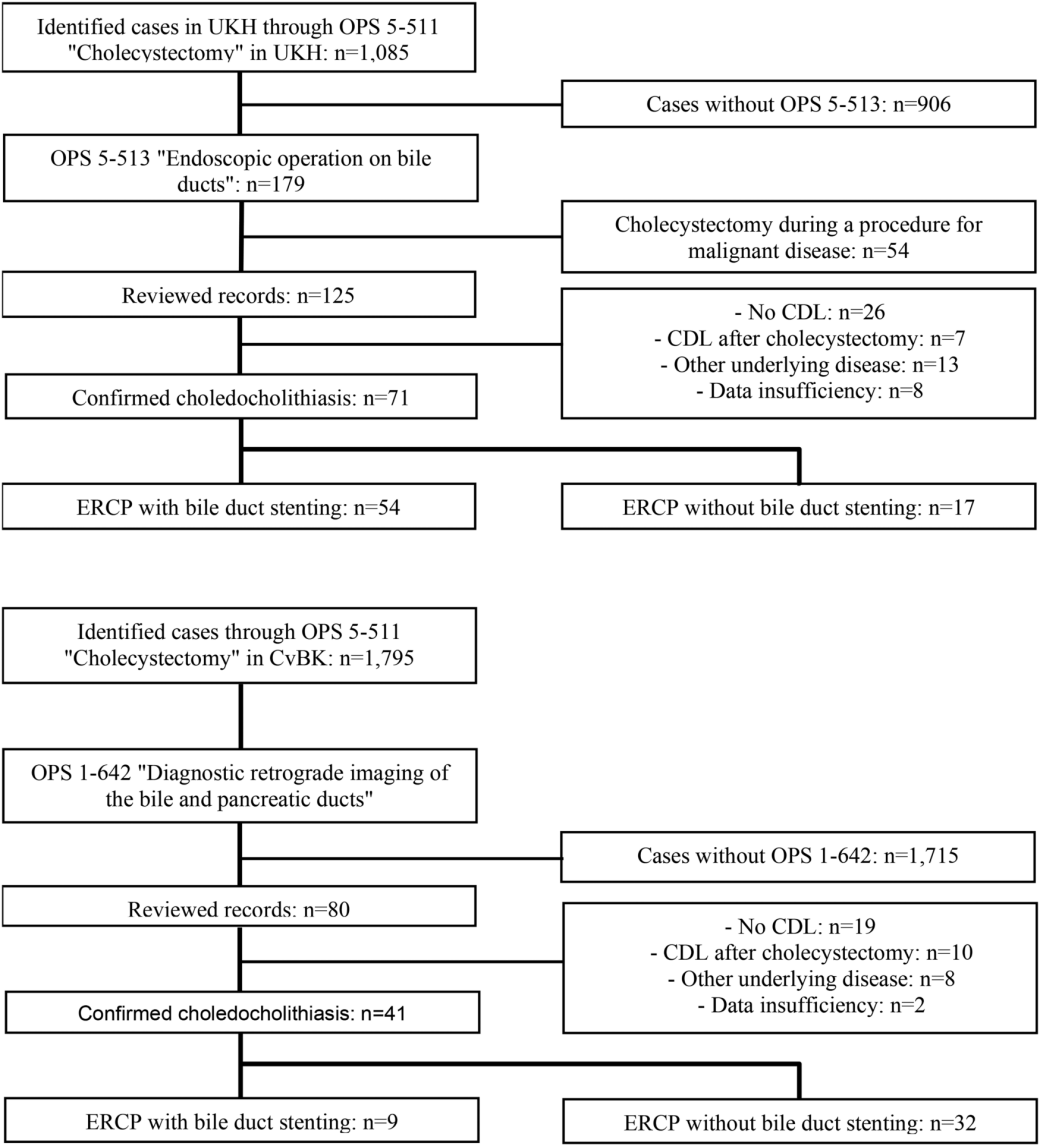
Flowchart depicting the patient selection process.

At UKH, the search for OPS code “5-511 Cholecystectomy” identified 1,085 cases (01.01.2016–31.12.2021) (Figure 1). After combining with “5-513 Endoscopic operation on bile ducts”, 179 potentially relevant cases remained, of which 54 were excluded due to underlying malignant conditions. After reviewing the remaining 125 records, another 54 cases were excluded based on the criteria mentioned above, leaving 71 patients with CDL included in the study. At CvBK, the search for “5-511 Cholecystectomy” yielded 1,795 cases for the same period. Using the additional criterion of “1-642 Diagnostic retrograde imaging of bile and pancreatic ducts”, the cohort was narrowed to 80 cases. After reviewing, 39 patients were excluded, and 41 were included. The reasons are presented in the flowchart (Figure 1). In total, 112 patients were included in the study cohort.

The total population (*N* = 112) had a median age of 68.5 years. In the group without stent, the proportion of women was 57.1% (*n* = 28), 9.5% higher than in the group with a stent (47.6%, *n* = 30) (*p* = 0.32). The two groups showed no significant differences in their median BMI [28.30 kg/m^2^ (25.92–31.14) vs. 27.75 kg/m^2^ (24.24–33.11); *p* = 0.77]. The distribution of patients with type 2 diabetes mellitus was also nearly equal between the groups (30.6%, 15/49) vs. 30.2% (19/63); *p* = 0.96). The proportion of patients who had undergone prior abdominal surgery was 16.8% higher in the stent group with 41.8% (23/63), compared to 24.5% (12/49) in the group without a stent (*p* = 0.07). A statistically significant difference was found between the two groups regarding the presence of cholangitis at the time of hospital admission. In the group without a stent, 24.5% (12/49) had cholangitis, while in the stent group, this proportion was 54% (34/63) (*p* = 0.002). Another significant difference was observed in the severity of illness as classified by the ASA score at the time of surgery. In the group without stenting, 29.2% (14/48) had an ASA classification greater than two, whereas in the stent group, this proportion was 60.3% (38/63) (*p* = 0.001). These data are summarized in Table 1.

**Table 1:** Study population characteristics.

Characteristic	Total (*N* = 112)	Without Stent (*n* = 49)	With Stent (*n* = 63)	*p*-value
Gender, *n* (%)				
Male	54 (48.2)	21 (42.9)	33 (52.4)	0.317
Female	58 (51.8)	28 (57.1)	30 (47.6)	
Age (years)	69 (51–78)	65 (48–78)	72 (75–80)	0.256
BMI (kg/m^2^)	28.07 (24.33–31.71)	28.30 (25.92–31.14)	27.75 (24.24–33.11)	0.765
Diabetes mellitus type 2, *n* (%)	34 (30.4)	15 (30.6)	19 (30.2)	0.959
Abdominal surgery, *n* (%)	35 (34.0)	12 (25.0) missing: 1	23 (41.8) missing: 8	0.072
Cholangitis on admission, *n* (%)	46 (41.1)	12 (24.5)	34 (54)	0.002
ASA-score >2	52 (46.4)	14 (29.2) missing: 1	38 (60.3)	0.001

Continuous variables given as median (Q1–Q3).

In the multivariable regression, prior surgeries (OR = 3.51 95% CI 1.24–10.08, *p* = 0.02), cholangitis at admission (OR = 3.02, 95% CI 1.10–8.27, *p* = 0.032), and bile duct diameter (OR = 1.16, 95% CI 0.996–1.34, *p* = 0.057) were identified as independent factors for stent implantation (Table 2).

**Table 2:** Influencing factors for stenting (multivariable binary logistic regression, method: backward elimination).

Variable	OR	95% CI	*p*-value
Prior abdominal surgery (yes)	3.513	1.224–10.084	0.020
Cholangitis at admission (yes)	3.018	1.101–8.272	0.032
Bile duct diameter (mm)	1.156	0.996–1.343	0.057

The goodness of fit was acceptable with a Nagelkerkes R-squared of 0.235.

Overall, 46 patients in the study cohort experienced complications of varying severity. The group with stenting had a complication rate of 52.4% (33/63), while the group without stenting had a complication rate of 26.5% (13/49) (*p* = 0.006) (Table 3).

**Table 3:** Comparison of clinical and procedural variables between patients with and without bile duct stenting: gender, age, BMI, type 2 diabetes mellitus, prior abdominal surgery, cholangitis on admission, abdominal ultrasound, endosonography, ERCP, MRCP, number of bile duct stones >5, bile duct diameter, gallstone diameter, CRP, leukocyte count, g-GT, bilirubin, AP, ALAT, and ASAT.

Variable	Without complications (*n* = 66)	With complications (*n* = 46)	*p*-value
Stenting of bile duct, *n* (%)			
Without	36 (73.5)	13 (26.5)	0.006
With	30 (47.6)	33 (52.4)	
Gender, *n* (%)			
Male	29 (53.7)	25 (46.3)	0.278
Female	37 (63.8)	21 (36.2)	
Age	66.0 (50.0–78.0)	74.0 (55.25–81.50)	0.096
BMI	28.39 (24.32–32.21)	27.57 (24.49–31.26)	0.547
Diabetes mellitus type 2, *n* (%)	21 (31.8)	13 (28.3)	0.687
Prior abdominal surgery, *n* (%)	16 (25.4)	19 (47.5)	0.021
Missing	3	6	
Cholangitis on admission, *n* (%)	22 (33.3)	24 (52.2)	0.046
Abdominal ultrasound, *n* (%)	66 (100)	46 (100)	–
Endosonography, *n* (%)	55 (83.3)	36 (78.3)	0.499
ERCP, *n* (%)	66 (100)	46 (100)	–
MRCP, *n* (%)	8 (12.1)	3 (6.5)	0.521
Number of bile duct stones >5, *n* (%)	17 (29.3)	13 (31.7)	0.798
Bile duct diameter, (mm)	10 (8–12)	11 (8.00–13)	0.326
Gallstone diameter, (mm)	5 (3.8–8)	6 (5–9.5)	0.197
CRP (mg/L)	8.8 (2.87–30.95)	41.2 (11.6–117.5)	0.003
Leukocyte count (Gpt/L)	9.24 (7.57–11.94)	9.82 (7.67–13.63)	0.208
g-GT (µkat/L)	6.48 (2.23–10.31)	6.23 (1.97–10.99)	0.831
Bilirubin (µmol/L)	39.05 (15.65–67.28)	50.9 (23.1–105.8)	0.328
AP (µkat/L)	2.60 (1.67–4.29)	3.09 (1.88–5.79)	0.263
ALAT (µkat/L)	3.04 (1.15–6.55)	1.88 (0.95–5.34)	0.346
ASAT (µkat/L)	2.95 (1.06–5.39)	2.49 (0.69–4.22)	0.241

Continuous variables given as median (Q1–Q3).

Wound healing disorders at 23.9% (*n* = 11), post-ERCP pancreatitis at 15.2% (*n* = 7), and postoperative delirium at 8.7% (*n* = 4) accounted for the majority of the complications.

There were 18 cases with preoperative complications (16.1% of the study population). Those included post-ERCP pancreatitis (6.3%, *n* = 7), infectious events such as cholangitis and cholangiosepsis 4.5%, *n* = 5), and ERCP-related complications like perforations, bleeding, or ileus (4.5%, *n* = 5). Two patients with infectious complications died due to sepsis and there was one case with acute kidney failure (0.9%). Among postoperative complications (*n* = 28, 25.0%), wound healing disorders were the most common (9.8%, *n* = 11). Surgery-related complications, such as bilioma, subhepatic abscesses, prolonged drainage secretion, or cystic stump insufficiency, appeard in 6.25% (*n* = 7) of the cases. Additionally, four cases of postoperative delirium (3.6%), two cases with postoperative cholangitis (1.8%) and two cases of stent dislocation (1.8%) were observed. There was one death due to sepsis, and another case was also fatal, although a direct cause could not be identified. The proportion of stenting is significantly lower in the group without complications than in the group with complications [45.5% (*n* = 30) vs. 71.7% (*n* = 33), *p* = 0.006].

The groups differed in the proportion of patients who had undergone prior abdominal surgery (*p* = 0.02); the group without complications had a proportion of 25.4% (16/63), while the group with complications had a proportion of 47.5% (19/40). In the group of patients with complications, the proportion of those admitted with cholangitis was higher at 52.2% (24/46) (*p* = 0.046) than in the group without complications, where it was 33.3% (22/66). The diagnostic measures at admission and their results showed fewer differences. Only the median CRP level on the day of admission differed significantly between the groups. The group without complications showed a slightly elevated median CRP of 8.8 mg/L (2.87–30.95), whereas the group with complications had significantly higher CRP values, with a median of 41.2 mg/L (11.6–117.5) (*p* = 0.003) (Table 3). The multivariable model after backward elimination (*n* = 101) confirmed these findings, showing that stenting (OR = 2.44, 95% CI 0.98–6.06, *p* = 0.06), prior abdominal surgeries (OR = 2.64, 95% CI 1.06–6.56, *p* = 0.04), and CRP levels (OR = 1.01, 95% CI 1.001–1.016, *p* = 0.023) were significant factors for the occurrence of overall complications.

In the study population, patients treated with a stent had a median overall length of stay (LOS) of 19 days (12.5–26) across 3 hospital stays (3–4). In contrast, patients without stenting were treated across two hospital stays with a median LOS of 11 days (7.5–15.5). The two groups differed significantly (*p* < 0.001). Furthermore, it was observed that the length of stay (LOS) increased with the occurrence of complications [with complications: 23 days (14–31.5) vs. without complications: 11 days (9–16); *p* < 0.001].

The combination of the aspects of stenting and complications shows that the length of stay (LOS) for patients with stenting is greater, regardless of whether complications occur, than for the group without stenting. There is a significant difference between the group without stenting and without complications, which had a median LOS of 10 days (6.25–12.75), and the group with stenting and without complications, which had a median LOS of 14 days (10–20.5) (*p* < 0.001). This difference is smaller in the group with complications, with a median difference of 6 days [without stenting: 18 days (10.5–25.5) vs. with stenting: 24 days (16.25–35.5); *p* = 0.06]. When viewed from the opposite perspective, it is evident that the occurrence of complications, regardless of stent placement, leads to an extended hospital stay (Table 4).

**Table 4:** Length of stay (LOS) based on stenting and complications.

Median LOS	Without stenting	With stenting	*p*-value
Without complications	10 d (6.25–12.75) *n* = 36	14 d (10–20.5) *n* = 29	*p* < 0.001
With complications	18 d (10.5–25.5) *n* = 13	24 d (16.25–35.5) *n* = 32	*p* = 0.060
*p*-value	0.002	<0.001	

Continuous variables given as median (Q1 – Q3). Mann–Whitney *U*-test.

The time interval between the first ERCP and the CCE showed a trend towards an increased risk of postoperative complications (OR = 1.004, 95% CI 0.999–1.009, *p* = 0.137). The median time interval was 41 days without a significant difference between the groups with and without stenting (*p* = 0.52).

The placement of a stent, regardless of whether complications occurred, was associated with higher reimbursement. A relative cost weight of 1.0 currently corresponds to approximately €4,200 in Saxony-Anhalt. In the group without complications, the median cost weight for a procedure with stenting was 2.739 (2.352–3.580), compared to the procedure without stenting at 1.838 (1.8–2.106), which represents a significant difference (*p* < 0.001). In the group with complications, patients with stenting also had a higher reimbursement with a relative cost weight of 3.783 (2.845–4.623). Patients treated without stenting had a lower median relative cost weight of 2.484 (1.954–3.417) (*p* = 0.004). Cases with complications were reimbursed at a higher rate, regardless of the use of stenting. In patients without stenting, the median relative cost weight for complications was 2.484 (1.954–3.417), while without complications it was 1.838 (1.8–2.106) (*p* = 0.003). The analogous comparison for patients with stenting shows a relative cost weight of 3.783 (2.845–4.623) when complications occurred, and a relative cost weight of 2.739 (2.352–3.580) without complications (*p* = 0.006). In summary, it can be observed that reimbursement rates were higher in the group with stenting, regardless of the occurrence of complications. Additionally, the occurrence of complications, independent of stenting, was associated with increased reimbursement (Table 5).

**Table 5:** Median revenues of the study population (divided by stenting and occurrence of complications).

Median relative cost weight	Without stent	With stent	*p*-value
Without complications	1.838 (1.8–2.106) *n* = 36	2.739 (2.352–3.58) *n* = 28	<0.001
With complications	2.484 (1.954–3.417) *n* = 13	3.783 (2.845–4.623) *n* = 29	0.004
*p*-value	0.003	0.006	

Continuous variables given as median (Q1 – Q3). Mann–Whitney *U*-test.

## Discussion

In this retrospective study we analyzed the clinical outcome of patients with CDL depending on bile duct stenting and consecutive cholecystectomy. Furthermore, we assessed variables influencing the clinical course and reimbursement.

We report three main findings in this study. First, bile duct stenting was associated with a significantly higher complication rate compared to the group without stenting. Second, hospitalization rate and overall LOS was significantly longer for patients with stenting. Third, reimbursement was significantly higher for patients with bile duct stenting, independently on the occurrence of complications.

Regarding the higher complication rates in the stenting group, our study aligns with findings from recent publications. Vandervoort et al. (27) reported a complication rate of 11.2% following ERCP, while Katsinelos et al. (28) observed a similar rate of 12%. In contrast, a significantly lower complication rate was noted in a meta-analysis by Cotton et al. (29), which included nearly 11,500 cases and reported a post-ERCP complication rate of 4%. More recently, Ak et al. (30) documented a post-ERCP complication rate of 20.3%. The complication rates observed in our study fall within the mid-range of these reported findings, despite different definitions across the studies.

The increased risk of stent-associated complications has to be balanced with the potential benefit of this procedure. In a randomized controlled trial evaluating the effect of prophylactic biliary stenting on reducing choledocholithiasis recurrence and biliary complications in patients awaiting cholecystectomy, 66 patients with biliary clearance were randomized into stenting and non-stenting groups. The recurrence of choledocholithiasis did not significantly differ between the stent and no-stent groups (20.6% vs. 9.4%, *P* = 0.306). However, patients in the stent group had a significantly higher complication rate, including cholecystitis and post-ERCP pancreatitis (*P* = 0.024). No patients in either group required emergency ERCP during follow-up (31). These findings, along with our own, suggest that prophylactic biliary stenting does not reduce choledocholithiasis recurrence and may, in fact, increase complication rates in this patient population.

The prolonged LOS observed in patients with stenting, along with increased costs regardless of complication rates, calls for a critical reassessment of the continued use of stents, particularly given the evidence linking them to higher complication rates (32, 33). One possible explanation is the common delay in performing laparoscopic cholecystectomy following ERCP. Current data do not support the superiority of delayed cholecystectomy over performing the surgery during the same hospital admission, which could potentially eliminate the need for stent placement, thereby reducing both costs and complication rates (34). Nevertheless, the practice of separating procedures for treating CDL across multiple hospital stays appears to be primarily driven by a desire to minimize economical harm rather than by a focus on generating profit through the performance of unnecessary procedures. This is because DRG reimbursement would not compensate for all procedures performed, such as CCE and ERCP, if they were to take place in one stay. However, stent placement introduces the need for an additional hospital stay for ERCP, raising the concern that financial incentives may influence its use.

Financial pressures on hospitals create a healthcare environment where patient care competes with economic considerations. One way out of this circle is the increasing rate of cholecystectomy and outpatient endoscopies including stent removal. Further rigorous studies are needed to determine the need for stenting and the optimal timing of cholecystectomy and its impact on both clinical outcomes and healthcare costs.

Our study has several limitations. The retrospective design poses a significant constraint, as it increases the potential for bias. The primary limitation is the small sample size, which may affect the generalizability of the findings. Furthermore, in most cases, stent removal occurred during a second ERCP after CCE, but exact timing varied due to retrospective design. It is important to emphasize that patients who received bile duct stents were more likely to present with more severe clinical conditions at baseline, such as cholangitis and higher ASA scores. These factors may have influenced both the decision to place a stent and the higher complication rates observed. Therefore, confounding by indication must be considered as a central limitation of the study. The observed differences in reimbursement are specific to the German DRG-based hospital reimbursement system and may not be generalizable to health systems in other countries with different economic models. Nevertheless, the overall trend toward increased resource utilization remains a relevant observation. Stenting was not formally protocolized during the study period and was left to the discretion of the endoscopist, often based on factors such as incomplete duct clearance, cholangitis, or perceived procedural risk. This variation reflects real-world practice but may limit external generalizability. In most cases, a second ERCP for stent removal was performed after the cholecystectomy. However, due to the retrospective nature of the study and lack of standardized follow-up protocols, the exact number of such staged procedures and their avoidability could not be reliably assessed. As a result, the findings should be interpreted and applied with caution. Furthermore, the groups with and without stents are not directly comparable, as certain patients had a more compelling clinical indication for stent placement from the outset, due to cholangitis, for example. Despite these limitations, the results offer valuable insights for clinicians managing this patient population.

Bile duct stenting in the management of choledocholithiasis is associated with increased complication rates, prolonged hospital stays, and higher costs. A more restrictive placement of biliary stents might be advisable. Furthermore, prioritizing early cholecystectomy when appropriate can help minimize complications and improve patient outcomes. A focused effort to implement these best practices in routine clinical care may lead to better short- and long-term treatment results.

## Data Availability

The raw data supporting the conclusions of this article will be made available by the authors, without undue reservation.
